# Performance Monitoring via Functional Near Infrared Spectroscopy for Virtual Reality Based Basic Life Support Training

**DOI:** 10.3389/fnins.2019.01336

**Published:** 2019-12-12

**Authors:** Emin Aksoy, Kurtulus Izzetoglu, Engin Baysoy, Atahan Agrali, Dilek Kitapcioglu, Banu Onaral

**Affiliations:** ^1^Department of Biomedical Device Technology, Acıbadem Mehmet Ali Aydınlar University, Istanbul, Turkey; ^2^Center of Advanced Simulation and Education, Acıbadem Mehmet Ali Aydınlar University, Istanbul, Turkey; ^3^School of Biomedical Engineering, Science and Health Systems, Drexel University, Philadelphia, PA, United States

**Keywords:** functional near infrared spectroscopy, basic life support, medical training, virtual reality, medical simulator

## Abstract

The use of serious game tools in training of medical professions is steadily growing. However, there is a lack of reliable performance assessment methods to evaluate learner’s outcome. The aim of this study is to determine whether functional near infrared spectroscopy (fNIRS) can be used as an additional tool for assessing the learning outcome of virtual reality (VR) based learning modules. The hypothesis is that together with an improvement in learning outcome there would be a decrease in the participants’ cerebral oxygenation levels measured from the prefrontal cortex (PFC) region and an increase of participants’ serious gaming results. To test this hypothesis, the subjects were recruited and divided into four groups with different combinations of prior virtual reality experience and prior Basic Life Support (BLS) knowledge levels. A VR based serious gaming module for teaching BLS and 16-Channel fNIRS system were used to collect data from the participants. Results of the participants’ scores acquired from the serious gaming module were compared with fNIRS measures on the initial and final training sessions. Kruskal Wallis test was run to determine any significant statistical difference between the groups and Mann–Whitney *U* test was utilized to obtain pairwise comparisons. BLS training scores of the participants acquired from VR based serious game’s the learning management system and fNIRS measurements revealed decrease in use of resources from the PFC, but increase in behavioral performance. Importantly, brain-based measures can provide an additional quantitative metric for trainee’s expertise development and can assist the medical simulation instructors.

## Introduction

Advances in new technologies allowed many domains to fulfill their changing needs with a new set of tools. Education is also one of those domains in which educators and learners are now using various serious gaming modules to meet different training requirements. Medical training professionals adopted these serious game based learning modules as medical simulation trainings and they have utilized it as a part of their curriculum. Previous studies have shown the advantages of interactive media tools over standard textbook lessons such as game based learning (Holzinger et al., 2008; Prensky, 2012; Deterding, 2013). Serious games also provide considerably positive effects on subject understanding, diligence and motivation (Pellas et al., 2018). Serious gaming modules can benefit different display technologies such as virtual reality (VR) head mounted displays, PC monitors and tablet PCs. Because of its high immersion levels, VR head mounted displays have attracted more interest for medical content development. Many instructors began to utilize VR-based approach in their teaching activities, due to the capacity of VR technologies to highly engage trainees through virtual environments and dynamic conditions (Hanson and Shelton, 2008; Holzinger et al., 2008; Prensky, 2012; Deterding, 2013). Serious gaming modules exploit scoring methods such as accuracy, task completion, and response times to assess the effectiveness of the serious gaming module and performance of the learner. However there is a need for an additional tool, enabling instructors to measure the efficacy of the modules and providing them an increased accuracy while assessing the real performance of the trainee.

It is well studied that task–specific activation of prefrontal cortex (PFC), is related with sensory, motor skills, and cognitive processes in human brain (Kelly and Garavan, 2005). These neural activities and mental workload during learning and practicing were successfully measured and quantified by using functional near infrared spectroscopy (fNIRS) over the last decade. fNIRS has been utilized as a functional brain activity monitoring technique in various field settings since it provides a safe, non-invasive, and practical method which measures the real time hemodynamic responses associated with brain activity changes. It is shown that, a particular amount of near infrared light can be transmitted for long distances throughout biological materials between the range of 700 and 1300 nm and additionally concentration differences of oxyhemoglobin (HbO_2_) and de-oxyhemoglobin (HHb) molecules (changes as small as 0.05–0.10 μM in tissues) in biological molecules shows different absorption amount of emitted infrared light (Jöbsis, 1977; Villringer, 1997; Maria et al., 2000).

Measured absorption and scattered amount of photons can be interpreted and assessed by using modified Beer–Lambert Law (Cope and Delpy, 1988; Chen et al., 1999; Rolfe, 2000). We have used the Oxy [oxygenation = OxyHb − deOxyH] and the localized activity changes which are known to be associated with working memory for this study (Bunce et al., 2006; Sato et al., 2011, 2013).

To date, fNIRS was exploited for assessment of operator’s cognitive performance different disciplines ranging from aviation to medical domain (Ayaz et al., 2012; Armstrong et al., 2018). In clinical settings, measurements of PFC activation by fNIRS while performing various surgical tasks (open surgical knot tying, navigational task, laparoscopic localization task, laparoscopic surgery task, robot assisted tasks, and etc.) were reported in many studies (Leff et al., 2007, 2008; Mylonas et al., 2008; Ohuchida et al., 2009; James et al., 2010, 2011; Shetty et al., 2016; Modi et al., 2017; Nemani et al., 2018).

As for the medical profession training, Basic Life Support (BLS) is a healthcare training course that assures responding to a patient correctly during cardiac and respiratory arrest. Smith et al. investigated major decline of BLS skills retention in 3, 6, 9, and 12 months, because of that, BLS courses are organized more frequently in most of the medical training centers (Smith et al., 2008). In this study, a VR based serious game module, 3D Medsim (Bochum/Germany), was run for teaching the algorithm of “BLS” training compatible with ERC (European Resuscitation Council) 2015 Guidelines (Perkins et al., 2015). Training induced mental workload and PFC activation of participants were quantified through fNIRS system while the participants are using the VR based BLS serious gaming module. Hypothesis of this study is to determine whether there is any decline in Oxy data as the participants learn and become familiar with both VR and BLS protocols. A significant reduction in the amount of PFC activation is expected with increased task familiarity and skill acquisition.

## Materials and Methods

### Participants

A total of 22 right handed subjects participated in this study classified into four different groups based on their knowledge level in BLS procedure and familiarization with VR games. These groups and number of participants within each group are listed on Table 1. This study has been reviewed and approved by the Ethical Committee of Acıbadem Mehmet Ali Aydinlar University. All participants gave written informed consent in accordance with the Declaration of Helsinki (World Medical Association, 2013).

**TABLE 1:** Groups and number of participants.

	**VR** **experience**	**BLS** **knowledge**	**Healthcare** **professional**	***N***	**%**
Group 1	−	−	No	7	31.8
Group 2	+	−	No	5	22.7
Group 3	+	−	Yes	5	22.7
Group 4	−	+	Yes	5	22.7
		Total		22	100.0

### Experimental Protocol

3DMedsim VR based BLS serious gaming software compatible with ERC 2015 BLS algorithm was used for the study (Perkins et al., 2015). The knowledge levels of the participants were assessed by the scoring system provided by the serious gaming software (Smith et al., 2008; Perkins et al., 2015; Aksoy, 2019). Following scores of the gaming module, denoted in Table 2, revealed the knowledge levels of the participants. Head mounted displays (HTC Vive) with high flickering rate and resolution were used in this study in order to minimize the potential risk of dizziness. The participants were also informed about this potential risk. At the beginning of the VR test, the subjects were asked to check around in VR platform in order to provide a transition moment for getting used to virtual environment. Following that the subjects were instructed about the correct actions to be taken and played the rescuer role interactively.

**TABLE 2:** Scoring criteria of the VR based serious game.

**Criteria**	**Points**
Checking consciousness	10
Head tilting	10
Checking breathing	10
Telling someone to call 911	10
Sending someone to fetch an AED (Automated External Defibrillator)	10
Controlling carotid pulse	10
Effective chest compression	10
Opening AED Device	10
Placement of AED pads	10
Defibrillation with AED	10
Total	100

Unlike tutorial module, in self-training tasks without hints or instructions to follow, subjects were expected to perform proper steps in an order within a correct timing. Self-training tasks were integrated with a Learning Management (LMS) scoring system providing assessment scores at the end of the session. BLS training protocol was based on 10 subsequent criteria as tabulated in Table 3 (Aksoy and Sayali, 2019).

**TABLE 3:** Basic life support algorithm based on ERC 2015 criteria.

**Criteria**
Ensure that the scene is safe
Check responsiveness by shaking gently and shouting loudly
Open the airway using the head tilt and chin lift technique
Telling someone to call 911
Sending someone to fetch an AED
Starting and continuing high-quality CPR in 30 compressions and 2 ventilations sequence
Attaching the AED pads when it arrives
Following the instructions are given by the AED
Delivering shock when advised by the AED
Continue with CPR for another 2 min or until the patient starts breathing

**FIGURE 1:**
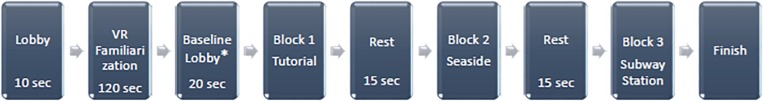
Experimental protocol work flow (^∗^ indicates starting point for people who had prior VR experience).

Experimental protocol delineated in Figure 1 starts with a lobby environment in VR, where participants are informed not to move for 10 s for the initial fNIRS baseline recording. Participants who had no prior VR experience had a VR familiarization session for 120 s. Familiarization session consist of basics tasks such as browsing within the software and using HTC Vive controller in VR environment. Participants who had completed the familiarization session directed to lobby screen. Participants who did not require a VR familiarization session started the game from lobby screen. Experiment had three blocks: “tutorial,” “seaside,” and “subway station.” After the completion of each block participants directed to lobby and rest for 15 s before starting the next block. “Tutorial,” which utilized a visual assistant, acted as an orientation and aimed to make users more familiar on how to perform BLS tasks. Upon completion of the “tutorial,” participants performed “seaside” and “subway station” blocks. Both “seaside” and “subway station” blocks had the same scenario workflow in different environments.

### Functional Near Infrared Spectroscopy

**FIGURE 2:**
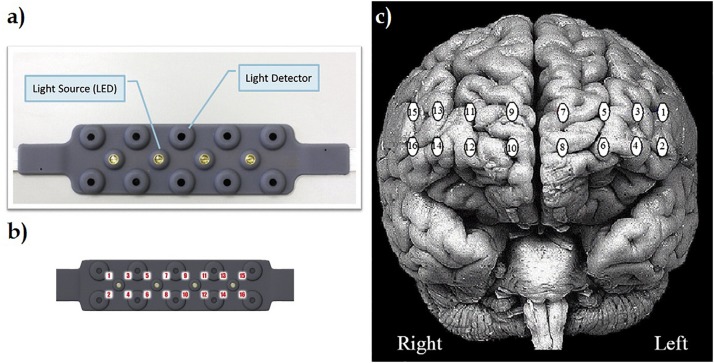
**(a)** fNIR sensor incorporates four light emitting diodes (LED) used as light sources, 10 light detectors. **(b)** Specific locations of entire 16 optode (channel) measurements over sensor pad. **(c)** Registration of 16 channels through PFC of brain surface (Ayaz et al., 2012).

A continuous wave fNIRS system (fNIR Devices, LLC, Potomac, MD, United States) was used in this study to monitor the hemodynamic response from PFC. The fNIRS system consists of three modules: a 16 channel sensor pad, a control box and a computer running data acquisition software. The sensor pad has four light emitting diodes (LED) used as light sources, 10 detectors. Entire 16 optode (channel) measurement locations are illustrated in Figure 2.

**FIGURE 3:**
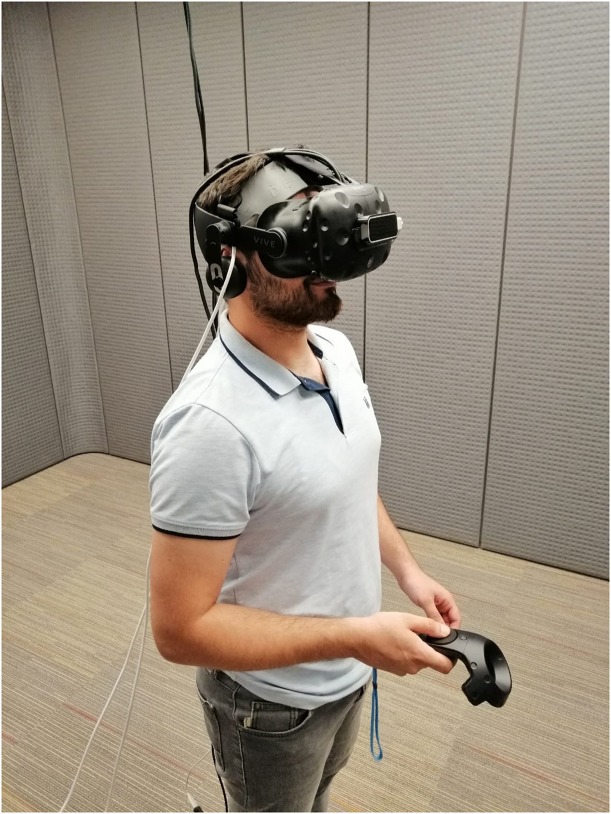
A subject wearing fNIRS pad and HMD.

The sensor pad was positioned on the PFC region of the participants (Figure 3). 730 and 850 nm wavelengths of lights were acquired continuously at the sampling rate of 2 Hz (Izzetoglu et al., 2007). fNIRS signal can be corrupted by instrument noise, physiological noise, and motion artifacts. Hence, to improve the sensitivity and spatial specificity of brain activity measures, a finite impulse response low pass filter was applied to hamper physiological confounding signals, such as respiration and heart beat oscillations. A linear phase low-pass FIR filter with cut-off frequency between 0.1 and 0.15 Hz has been used. The high deviations and motion artifact per channel were removed using the sliding-window motion artifact rejection reported in Ayaz et al. (2010). The filtered light intensity data were processed with the Modified Beer Lambert Law to calculate oxygenated (oxyHb) and deoxygenated hemoglobin (deoxyHb) values for each channel. Using these values, oxygenation measures (Oxy = OxyHb − DeoxyH) were derived, and left PFC region (Channel 3), known to be associated with working memory, was used within the scope of this study (Kelly and Garavan, 2005; Leff et al., 2008; Ayaz et al., 2012). Therefore, channel 3 was defined as the region of interest in this study. Since the sample size is limited, normality assumption cannot be provided and parametric methods cannot be used. Standard non-parametric tests have been used when assumptions of parametric tests cannot be achieved or the sample size is limited. One of the most common non-parametric test is Kruskal–Wallis test. Kruskal Wallis non-parametric method compare the distributions between groups. Significant result of a non-parametric test does not differentiate whether the difference is between the location and shape of the distributions. Thus, it limits the use of non-parametric tests especially where the shape of distribution between groups is very different (Dwivedi et al., 2017). In addition, the Shapiro Wilk test *p* value for each group was found to be greater than 0.05 in terms of OXY levels and game scores. All statistical analysis was carried out by using MedCalc Statistical Software version 12.7.7 (MedCalc Software bvba, Ostend, Belgium) (Schoonjans et al., 1995).

## Results

### Behavioral Performances

**FIGURE 4:**
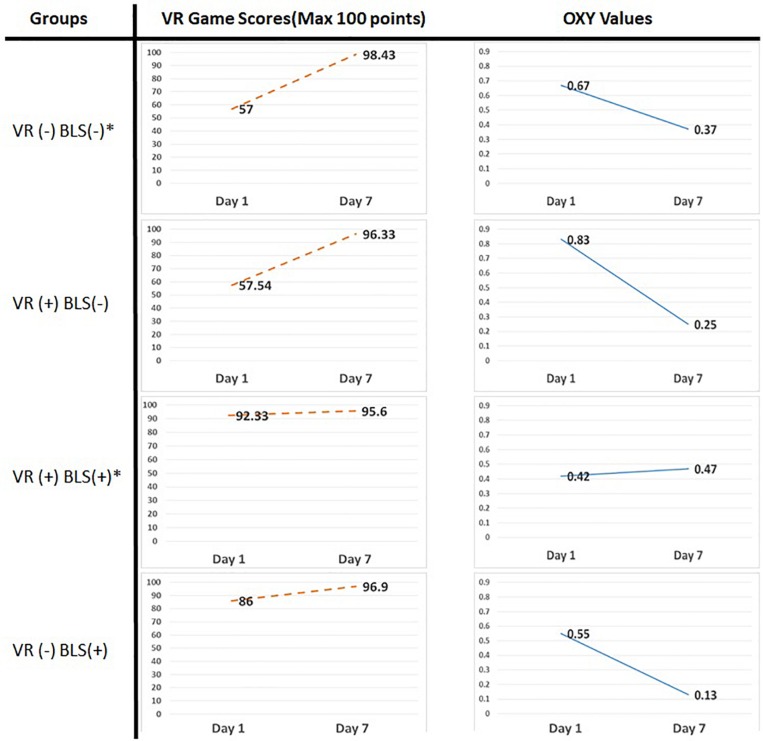
Serious gaming scores (column 1) of the subjects acquired from the learning management system of the game module on the first and seventh day and mean oxy values (column 2) from four groups on the first and seventh day of the study (^∗^ indicates statistical significant values) (Supplementary Table S1).

Basic life support scores for each group and sessions were calculated by the scoring system of the VR based serious gaming module (Figure 4). In all groups except group 3 (With Prior VR experience/prior BLS Knowledge) there is a statistically significant difference between groups for score in the first day (Kruskal Wallis *p* < 0.05). The improvement of BLS scores between the first and seventh day for all the groups can be seen on Figure 4. There is a significant improvement of BLS scores in group 1 (+72.68%) and group 2 (+67.42%), whereas there was no significant improvement of BLS scores in group 3 (+ 3.53%) and group 4 (+11.67%) (Table 4).

**TABLE 4:** Basic life support scores of four groups.

**Groups**	**VR−BLS−**	**VR+ BLS−**	**VR+ BLS+**	**VR− BLS+**
Day 1 Mean BLS Score	57.00	57.54	92.33	86.00
Day 7 Mean BLS Score	98.43	96.33	95.60	96.90
Improvements (%)	+ %72.68	+ %67.42	+ %3.54	+ %12.67

### fNIRS Results

Mean Oxy values per channel were calculated for each group to conduct a correlational analysis between subject’s training performances and cognitive workload assessed via the changes in blood oxygenation levels from the PFC region of human brain.

The oxy values of the healthcare professionals with prior VR experience remains almost the same in the first and seventh day (Figure 4). On the contrary, significant decreases of fNIRS oxy values can be seen in all other groups on the seventh day. The significant decreases of fNIRS Oxy levels on the seventh day were calculated by 54.90% in group 1, 30.47% in group 2, and 22.81% in Group 4.

Kruskal Wallis test was utilized to determine any significant difference between the groups and Mann–Whitney *U* test was utilized to obtain pairwise comparisons. The statistical analysis results are shown on Tables 5, 6.

**TABLE 5:** Statistical analysis of fNIRS data and serious gaming scores (100/100) by using Kruskal Wallis test.

**Groups**					

	**VR−, BLS−**	**VR+, BLS−**	**VR+, BLS+**	**VR−, BLS+**	***p***
	
	**Mean ± SS**	**Mean ± SS**	**Mean ± SS**	**Mean ± SS**	
fNIRS OXY Level Day 1	0.67 ± 1.18	0.82 ± 1.38	0.41 ± 1.09	0.55 ± 1.1	0.898
fNIRS OXY Level Day 7	0.37 ± 0.81	0.25 ± 0.38	0.47 ± 0.86	0.13 ± 0.49	0.879
Serious Game Day 1 Score	57 ± 21.7	54.2 ± 26.3	92.9 ± 7.3	85.9 ± 10.5	<0.001
Serious Game Day 7 Score	98.3 ± 2.9	96.4 ± 2.9	96.2 ± 47	96.9 ± 3.4	0.176

**TABLE 6:** *Post hoc* test results between pairwise comparisons of scores on day 1 by using Mann–Whitney *U* test (Supplementary Table S2).

	**VR− BLS−**	**VR− BLS−**	**VR− BLS−**	**VR+ BLS−**	**VR+ BLS−**	**VR+ BLS+**
	**VS.**	**VS.**	**VS.**	**VS.**	**VS.**	**VS.**
	**VR+ BLS−**	**VR+ BLS+**	**VR− BLS+**	**VR+ BLS+**	**VR− BLS+**	**VR− BLS+**
Pairwise comparison of performance scores (*p* values)	0.835	<0.001	<0.001	<0.001	<0.001	0.037

As shown on Table 5, there is statistically significant difference between groups for score in the first day (Kruskal Wallis *p* < 0.05). According to the *post hoc* test results, there is significant difference between all pairwise comparisons except VR−, BLS− and VR+, BLS− (Bonferroni correction *p* < 0.008 Mann–Whitney *U*). These statistical data reveal that prior VR experience had no additional positive effect on gaming scores.

## Discussion

The positive effect of tablet based and VR based serious gaming modules for healthcare was shown in various studies. Virtual gaming is becoming a part of the existing trainings programs for healthcare. Utilizing serious gaming as a self-learning strategy is time saving for both learners and educators, as serious gaming modules can be available by learners at any time or anywhere. The other advantages provided by serious gaming is that it improves decision making capabilities of learners in complex situations and self-learning capabilities by providing the learners with the opportunity to practice as much as they can (Johnson et al., 2016; Verkuyl et al., 2017; Kinder and Kurz, 2018).

The gaming module used for this study has a scoring system linked with a dedicated learning management system, allowing us to assess learners’ results in an objective manner. In current use of VR based gaming modules, there are still challenges about the technology of assessment including high-stakes assessment in medical education (Christopoulos et al., 2018). Due to similar needs for assessment, we were in a search for an additional tool for the assessment of VR based gaming modules.

To date, fNIRS was used in clinics for examination of many neuronal activities of infants and adults suffer from visual, auditory, olfactory, and mental diseases (Meek et al., 1998; Sakatani et al., 1999; Bartocci et al., 2000; Zaramella et al., 2001; Peña et al., 2003; Wilcox et al., 2005; Arenth et al., 2007). Psychiatric applications also benefit fNIRS imaging for examination of task-dependent abnormalities in PFC of patients with schizophrenia, depression and Alzheimer’s diseases (Fallgatter et al., 1997; Fallgatter and Strik, 2000; Matsuo et al., 2002; Suto et al., 2004; Kuwabara et al., 2006). fNIRS imparts a novel objective metric for better understanding of task specific motor skill assessment during surgical training certification as well. Based on these studies, fNIRS was chosen in this study as the tool for measuring the PFC activities and cognitive workload of the participants.

In our study, it has been found out that prior BLS knowledge had positive effects on both BLS scores and fNIRS values. There was a statistically significant difference in gaming scores between the groups on the first day. The improvement of BLS gaming scores for groups 1 and 2 (without prior BLS knowledge) between the first and seventh day was detected. These values reveal that the VR based serious gaming module used for this study had a positive effect on learning the content of BLS for the groups without prior BLS knowledge. Because of the tutorial session with VR equipment at the initial phase of the study, it is observed that having no prior VR experience did not have a significant effect on the gaming scores and fNIRS results. Limitations in this study were the risk of dizziness due to HMD displays and discomfort during the training sessions caused by the cables of both HMD displays and the fNIRS head pad. For future studies, we plan to benefit wireless HMD displays with the same or higher resolution of the ones used and wireless fNIRS systems allowing the operators to move more freely with less discomfort during the trainings. A limitation of this study was the limited number of subjects but this study focuses on the overall effect measurement, and within the scope of this exploratory study, total number of subjects (*n* = 22) is considered sufficient for the effect size. It is also important that it is difficult to have an access to professional healthcare staff with prior VR experience and BLS knowledge for an exploratory research study.

The decrease of mean fNIRS levels in all groups except the expert group (Group 3) may indicate that the cognitive workload of the participants were decreased once they became familiar with the task and had enough practices at the seventh day of the study. These findings are in agreement and supported by the previous studies (Ayaz et al., 2012). The correlation between the fNIRS data and gaming scores reveal that fNIRS can be used as a complementary technological tool for assessment in addition to the behavioral performance via serious gaming modules.

## Conclusion

Emergency situations are encountered frequently in the healthcare environment and it is crucial for the expert healthcare providers to be ready for the distraction factors caused by potential emergency situations. Besides scores acquired from simulators’ or serious gaming modules’ embedded scoring systems, measuring mental/cognitive workload during these trainings can be a complimentary tool to assess the readiness of healthcare workers to perform their duties during emergency situations. Training scores acquired from VR based BLS serious game and fNIRS Oxy level measurements of the participants reveal that fNIRS can be used as an additional tool for assessment supporting medical simulation educators by monitoring cognitive workload during training. Utilizing fNIRS measurements, we were able to reveal a significant reduction in the amount of PFC activation with increased task familiarity and skill acquisition. Due to these promising results, we plan to combine fNIRS measurements with scoring algorithms of other serious gaming modules and medical simulation modalities in our future studies.

## Data Availability Statement

All datasets generated for this study are included in the article/Supplementary Material.

## Ethics Statement

The studies involving human participants were reviewed and approved by the Acıbadem Mehmet Ali Aydınlar University Ethical Committee (ATADEK). The patients/participants provided their written informed consent to participate in this study.

## Author Contributions

EA: study design, VR module creation, and assessment. KI and BO: study design and analysis of results. AA: fNIRS measurements. EB: data collection. DK: analysis of results.

## Conflict of Interest

fNIR Devices, LLC manufacturers the optical brain imaging instrument and licensed IP and know-how from Drexel University. KI and BO were involved in the technology development and thus offered a minor share in the startup firm, fNIR Devices, LLC.

The remaining authors declare that the research was conducted in the absence of any commercial or financial relationships that could be construed as a potential conflict of interest.

## Supplementary Material

TABLE S1 Mean oxy values of channel 3 and serious gaming scores of the subjects acquired from the learning management system of the game module.

TABLE S2 Pairwise comparison (*p* values) between groups.
